# Complications and Success Rate of Percutaneous Nephrolithotomy in Renal Stone: A Descriptive Cross-sectional Study

**DOI:** 10.31729/jnma.4723

**Published:** 2019-12-31

**Authors:** Robin Joshi

**Affiliations:** 1 Department of Urology, Kathmandu Medical College and Teaching Hospital, Sinamangal, Kathmandu, Nepal

**Keywords:** *percutaneous nephrolithotomy*, *postoperative complications*, *renal calculi*

## Abstract

**Introduction:**

Renal stone disease has been affecting people for centuries. Percutaneous nephrolithotomy is one of the five interventions offered to a patient with renal stone. With the continuous development of noninvasive or minimally invasive techniques, these surgical procedures have been refined over time. This study was conducted to find the success rate of percutaneous nephrolithotomy in renal stone using Guy's score and complication by Modified Clavien score.

**Methods:**

This descriptive cross-sectional study was done among 114 patients who underwent percutaneous nephrolithotomy in a tertiary care hospital, from September 2016 to December 2018 after receiving ethical approval from the Institutional Review Committee. Convenient sampling was done. All patients were informed about the potential benefits and risks of the percutaneous nephrolithotomy procedure and patients signed an informed written consent form. Point estimate at 95% Confidence Interval was calculated along with frequency and proportion. Statistical analysis was done by using Statistical Package for Social Sciences version 22.2.

**Results:**

Forty-six (40.3%) patients had Guy's stone score I, 43 (37.71%) patients had a score of II, 15 (13.6%) patients had a score of III and 10 (8.77%) patients had a score of IV. The success rates of stone clearance were 97.8 %, 95.3%, 80% and 50% for Guy's stone score 1, 2, 3 and 4 respectively. A total of 114 patients were enrolled in the study out of which 66 were male and 48 were female. Eighteen patients experienced some form of complications out of which 3 patients needed surgical intervention with Modified Clavien score of III.

**Conclusions:**

Using Guy's scoring system for percutaneous nephrolithotomy we evaluated the success rate. It is reproducible, easy and proves to be a useful tool to counsel patients about stone-free rate and prognosis for the surgical procedure. Modified Clavien score was helpful in evaluating complication rate.

## INTRODUCTION

Renal stone disease has been affecting people for centuries. Individuals requiring intervention are offered mainly 1 of 5 interventions: ureteroscopic lithotripsy (URSL), extracorporeal shockwave lithotripsy (ESWL), percutaneous nephrolithotomy (PCNL), open surgery or retrograde intrarenal surgery (RIRS). Percutaneous removal of stones is currently recommended for patients with staghorn calculi, kidney stones greater than 2 cm, and lower pole stones greater than 1.5 cm.^1^

Complications during or after PCNL may be present with an overall complication rate of up to 83%.^2^ However, no standardized method is available to predict the stone-free rate after PCNL. Aiming to obtain a quick, simple and reproducible method for the prediction of PCNL outcomes, Thomas et al. developed Guy's scoring system to grade PCNL stone-free rate and complications.^3^ Scoring systems help a surgeon in choosing surgical options and bring about better patient outcome.

This study was conducted to find the complications and success rate of percutaneous nephrolithotomy in renal stone using Guy's score.

## METHODS

This was a descriptive cross-sectional study done at the Department of Surgery, Kathmandu Medical College Teaching Hospital (KMCTH) from September 2016 to December 2018. Convenient sampling method was used. Ethical clearance was obtained from the Institutional Review Committee of KMCTH. All patients were informed about the potential benefits and risks of the PCNL procedure and patients signed an informed written consent form. Convenient sampling method was used. Statistical analysis was done by using Statistical Package for Social Sciences.

Convenient sampling was done and the sample size was calculated using the formula,

$$\begin{array}{ll} \text{n} & ={\text{Z}}^{\text{2}}\times \text{(p}\times \text{q)}/{\text{e}}^{\text{2}}\\ & =\text{1}{\text{.96}}^{\text{2}}\times \text{0.5}\times (1-\text{0.5)}/\text{0}{\text{.1}}^{\text{2}}\\ & =96 \end{array}$$

where,

n= required sample size
p= prevalence of study (50%)
q= 1-p
e= margin of error, 10%
Z= 1.96 at 95 % CI

The calculated minimum sample size was 96, but the total sample taken was 114. All cases undergoing PCNL in KMCTH were included in the study with the exclusion of patients who did not give consent, the cases converted to open surgery, radiolucent stones, pregnant women and with a severe comorbid medical condition and with bleeding disorders.

Creatinine, bleeding and coagulation profile, and urine cultures were obtained from all patients. The radiologic evaluation was done using X-ray of Kidney Ureter and Bladder (KUB) or Intravenous urography (IVU) and/ or computed tomography intravenous urogram (CT IVU) and ultrasonography of KUB and/or Non-Contrast Computerized Tomography (NCCT) if needed. The stone burden was determined by radiographic studies, and patients were classified using the Guy's Stone Score (GSS) as Guy's 1, 2, 3 and 4.

PCNL was recommended for symptomatic patients with a total renal stone burden >1.5 cm or lower pole stones >10 mm. Patients underwent PCNL as per the standard protocol of our hospital after ensuring sterile urine. Antibiotic prophylaxis was given to all the patients. A nephrostomy tube was placed into the renal pelvis at the end of the procedure and tubeless as indicated.

Plain film of the kidneys, ureters, and bladder was obtained on the first postoperative day (POD). If complete stone clearance (less than 4 mm) was documented and the urine is not significantly hematuric, the nephrostomy tube was removed after 48-72 hours. After 48 hours, if there was no urine leak from the nephrostomy site, the urethral Foley's and ureteral catheters were removed. Double J (DJ) stent if placed was removed after 2-4 weeks and 4-6 weeks if there was an iatrogenic injury to the renal pelvis.

Data including the age, sex, and stone complexity score according to GSS, post-operative stone clearance, and duration of surgery was noted. The postoperative stone-free rate was determined at first POD by KUB radiogram. Statistical Analysis was made by using Statistical Package for Social Sciences version 22.2.

## RESULTS

A total of 114 patients were enrolled in the study out of which 66 (57.9%) were male and 48 (42.1%) were female. Mean age of the patients was 37.08. Their mean weight was 62.28±7.32 kg. Among the patients who were enrolled, 3 (2%) were underweight, 51 (44%) had normal BMI, 55 (48%) were overweight and 7 (6%) were pre-obese. Out of 114 patients, 74 (65%) patients had single stone and 40 (35%) had multiple stones. Seventy (61.4%) patients had concomitant hydronephrosis. The mean size of the stone was 2.49± 0.37 cm. Fifty nine patients had stone on the left side and 55 patients had it on the right side. Out of the total patients, the most common site of stone was in the pelvis followed by lower calyx (Table 1).

**Table 1:** Demographic profile and clinical parameters.

Variable	Value n (%)	Mean
Gender (n=114)		
Male	66 (57.9%)	
Female	48 (42.1%)	
Age Distribution		
10-19	4 (3.5%)	
20-29	28 (24.5%)	
30-39	43 (37.7%)	37.08
40-49	22 (19.3%)	
50-59	9 (7.8%)	
60-69	8 (7.01%)	
BMI		
Underweight(<18.5)	2 (2%)	
Normal(18.5-22.9)	50 (44%)	22.70
Overweight(23 - 24.9)	55 (48%)	
Pre obese(25-29.9)	7 (6%)	
No. of Stone		
Single	74 (64.9%)	
Multiple	40 (35.1%)	
Stone Size(cm)		
1-1.5	20 (17.5%)	
1.6-2	26 (22.8%)	2.496
2.1-2.5	43 (37.7%)	
2.6-3.5	15 (13.15%)	
3.5-3.9	10 (8.77%)	
Side of stone		
Right	55 (48.2%)	
Left	59 (51.8%)	
Hydronephrosis		
Present	70 (61.4%)	
Not present	44 (38.6%)	

**Figure 1:**
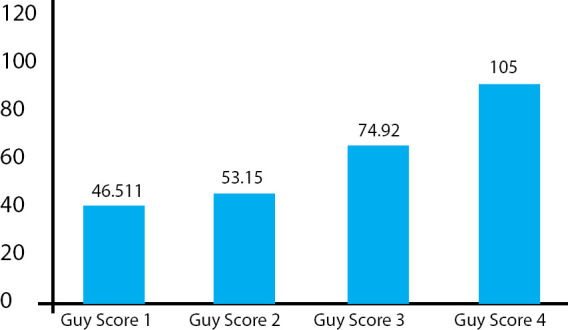
Mean Operative time (in minutes) according to Guy's scoring.

The mean operating time was 46.37, 53.9, 74.26, 105 minutes in Guy's score I, II, III and IV, respectively (Figure 1). Forty-six (40.3%) patients had Guy's stone score 1, 43 (37.71%) patients had a score of 2, 15 (13.6%) patients had a score of 3 and 10 (8.77%) patients had a score of 4 (Table 2).

**Table 2:** Guy's stone score among patients with stone clearance.

S.N.	Grade	n (%)	Stone clearance rate (%)
1.	1	46 (40.3)	97.8%
2.	2	43 (37.71)	95.3%
3.	3	15 (13.6)	80%
4.	4	10 (8.77)	50%

Nine patients developed urinary tract infection (3 in Guy's score 1 and two each from Guy's score 2, 3, 4). They were treated conservatively by upgrading antibiotics and later changing antibiotics according to urine culture sensitivity reports. Three patients developed postoperative atelectasis in Guy's score 2 and were treated with chest physiotherapy and incentive spirometry. Two patients developed pneumonia in Guy's score 3, and another with Guy's score 4 which was treated by upgrading antibiotics and later changing it according to sputum culture sensitivity reports. Two patients with Guy's score 3 had iatrogenic peritoneal leak following renal pelvis tear which was managed by intraperitoneal pigtail insertion per-operatively. One patient with guys score 3 developed stress peptic perforation and was treated accordingly. Eighteen patients experienced some form of complications out of which 3 patients needed surgical intervention with Modified Clavien score of III. Complication rate were 19%, 13.9%, 20% , 0% according to Modified Clavien I,II,III,IV for GUY'S stone score (Table 3).

**Table 3:** Complications according to the Modified Clavien scale.

	ClavienI (19%)	ClavienII (13.9%)	ClavienIII (20%)	ClavienIV
Guy's Score I	3	0	0	0
Guy's Score II	2	3	0	0
Guy's Score III	2	2	3	0
Guy's Score IV	2	1	0	0

The success rate of stone clearance according to Guy's stone score was 97.8 %, 95.3%, 80% and 50% for Guy's score 1, 2, 3 and 4 respectively (Table 2). Three patients with Guys scoring 3 underwent ESWL for remaining stone. For Guy's scoring 4, 3 patients had second session PCNL through the same tract to clear the stone on the second postoperative day. Remaining two patients needed only ESWL to clear the stones.

## DISCUSSION

The prevalence of urolithiasis around has increased over the past two decades, reaching nearly 7% in women and 10.3% in men.^3^ Though most of the smaller stones pass spontaneously or are amenable to shock wave lithotripsy, some stones will have to be removed surgically. The Guy's Stone Score (GSS), the Clinical Research Office of the Endourological Society (CROES) nomogram, S.T.O.N.E. (stone size, tract length, obstruction, number of involved calices, and essence/ stone density) nephrolithometry and Seoul National University Renal Stone Complexity (S-ReSC) score permit for impartial valuation of nephrolithiasis and results of PCNL.^4-7^ However, a study found no standardisation for the measurement of stone dimensions, tract length, Hounsfield units, and staghorn definition.^8^

The Guy's scoring system with other scoring systems can help improve patient care by helping the clinician in choosing surgical options and better patient counseling of stone clearance and prognosis. It would also enable clinicians to objectively assess and compare different technical modifications (e.g., supine vs. prone).^9^ In two different studies conducting external validation of the Guy scoring system, Mandal and Ingirmasson evaluated this scoring system as an efficient instrument in predicting the stone-free status.^10-11^

The appropriate method of predicting the outcomes after PCNL would be a scoring system that is quick, simple, uncomplicated, reproducible and has a good association with the SFR and complication rate. Thomas et al. studied a series of 100 consecutive PCNL procedures performed at their tertiary referral hospital by 3 endourologists. The distribution of cases according to the stone score was 28% classified as grade 1, 34% as grade 2, 21% as grade 3, and 17% as grade 4. The stone-free rate (SFR) was 62% of the completed procedures ranging from 81% for grade 1 to 29% for grade 4.^3^ In their series involving 278 PCNL cases, Mandal et al. observed that the stone-free rates decreased as 100%, 74%, 56%, and 0% according to the GS 1, 2, 3, 4 scores respectively.^11^

PCNL was started some 8 years back in 2010 at our centre in selected cases. With the gain of experience complicated cases with Guy's score of 3 and 4 were attempted. Therefore we conducted a stone clearance survey along with complication rate.

In our study, the most common site of stone was in the pelvis followed by lower calyx. Forty-six patients (40.3%) had Guy's stone score 1, 43 (37.71%) had a score of II, 15 (13.6%) had a score of III and 10 (8.77%) had a score of IV. In a study by Kumsar et al, of 122 patients who underwent PCNL, 75.5% of the patients were GS 1, 21.6% GS 2 and 2.9% GS 3. In their study, 10% of GS 1, 4% of GS 2 and 66% of GS 3 patients had residual stone.^12^ Eighteen patients experienced some form of complications out of which 3 patients needed surgical intervention with Modified Clavien score of III. We observed in our study that there was a positive correlation with the rate and severity of complication with Guy's scoring. Higher the GUY'S stone score more were the complication and less stone free rate as well. Since this is a single center study, the outcome might not be generalized. Real time fluoroscopy after the surgery and X-ray KUB was done to evaluate the stone clearance. XR KUB has relatively less sensitivity and specificity compared to that of CT KUB to evaluate the stone clearance.

## CONCLUSIONS

Modified Clavien complication scale is an effective tool in recognizing grade of complication. Guy's scoring system for PCNL is reproducible, easy and proves to be a useful tool to counsel patients about stone-free rate and prognosis for the surgical procedure.

## Conflict of Interest

**None.**
